# The contribution of lot-to-lot variation to the measurement uncertainty of an LC-MS-based multi-mycotoxin assay

**DOI:** 10.1007/s00216-018-1096-5

**Published:** 2018-05-01

**Authors:** David Stadler, Michael Sulyok, Rainer Schuhmacher, Franz Berthiller, Rudolf Krska

**Affiliations:** Center for Analytical Chemistry, Department of Agrobiotechnology (IFA-Tulln), University of Natural Resources and Life Sciences, Vienna (BOKU), Konrad-Lorenz-Straße 20, 3430 Tulln, Austria

**Keywords:** Relative matrix effect, Uncertainty budget, Recovery, Matrix mismatch, Proficiency test, Fit for purpose

## Abstract

Multi-mycotoxin determination by LC-MS is commonly based on external solvent-based or matrix-matched calibration and, if necessary, the correction for the method bias. In everyday practice, the method bias (expressed as apparent recovery RA), which may be caused by losses during the recovery process and/or signal/suppression enhancement, is evaluated by replicate analysis of a single spiked lot of a matrix. However, RA may vary for different lots of the same matrix, i.e., lot-to-lot variation, which can result in a higher relative expanded measurement uncertainty (*U*_*r*_). We applied a straightforward procedure for the calculation of *U*_*r*_ from the within-laboratory reproducibility, which is also called intermediate precision, and the uncertainty of RA (*u*_*r*,*RA*_). To estimate the contribution of the lot-to-lot variation to *U*_*r*_, the measurement results of one replicate of seven different lots of figs and maize and seven replicates of a single lot of these matrices, respectively, were used to calculate *U*_*r*_. The lot-to-lot variation was contributing to *u*_*r*,*RA*_ and thus to *U*_*r*_ for the majority of the 66 evaluated analytes in both figs and maize. The major contributions of the lot-to-lot variation to *u*_*r*,*RA*_ were differences in analyte recovery in figs and relative matrix effects in maize. *U*_*r*_ was estimated from long-term participation in proficiency test schemes with 58%. Provided proper validation, a fit-for-purpose *U*_*r*_ of 50% was proposed for measurement results obtained by an LC-MS-based multi-mycotoxin assay, independent of the concentration of the analytes.

**Electronic supplementary material:**

The online version of this article (10.1007/s00216-018-1096-5) contains supplementary material, which is available to authorized users.

## Introduction

Mycotoxins are toxic secondary metabolites produced by fungi on agricultural commodities and processed foods [1]. They have adverse effects on humans and animals that result in illnesses and economic losses. To protect the consumer from harmful effects, regulations limit maximum levels of mycotoxins in food and feed [2, 3]. Reliable analytical methods are needed for compliance testing and to monitor mycotoxin contamination.

Multi-mycotoxin analytical methods allow timely routine screening for a multitude of analytes with a broad spectrum of physiochemical properties [4]. Multi-mycotoxin methods are based on liquid chromatography electrospray ionisation tandem mass spectrometry (LC-ESI-MS/MS) [5–7] in combination with an extraction procedure that recovers a broad range of analytes [8]. In most cases, raw extracts are diluted and injected with limited [9] or even no sample clean-up [4, 5, 10], i.e. “dilute and shoot”, as clean-up steps would remove some of the analytes for further analysis. For multi-mycotoxin assays, quantification is commonly based on external solvent-based or matrix-matched calibration. In essence, the response of the analyte is compared to the calibration curve and, if necessary, is corrected for the method bias. The method bias, expressed as apparent recovery (RA), may be caused by losses during the recovery procedure (RE) or signal suppression/enhancement (SSE) [11]. Compensation of RA could be achieved by using a stable isotope-labelled internal standard. However, this approach is limited to mycotoxins where ^13^C-labelled internal standards are available and may not be feasible in economic terms.

A measurement result is only complete when it is accompanied by a statement of the associated uncertainty. Although various guideline documents on uncertainty estimation are available [12–17], an uncertainty statement is missing in most validation studies of multi-mycotoxin methods. For multi-mycotoxin methods, the relative expanded measurement uncertainty (*U*_*r*_) may be estimated from the uncertainty associated with the precision and the uncertainty associated with the method bias (if a bias correction is applied).

Especially when a bias correction is performed (e.g. analysis of patulin and aflatoxins in foodstuffs [18, 19]), the uncertainty of RA may be underestimated since the uncertainty contribution originating from bias is usually not considered. In everyday practice, RA is evaluated based on replicate analysis of a single lot of a matrix [4, 5, 8–10]. Due to the heterogeneous nature of food matrices, RA may vary for different lots (quantity of material known to have uniform characteristics such as origin and variety) of the same matrix resulting in “lot-to-lot variation” [20]. This so-called matrix-mismatch phenomenon would contribute to the uncertainty of RA and thus to *U*_*r*_ [21]. Matuszewski et al. first described differences in SSE for an analyte in plasma samples from different sources, which is referred to as *relative* matrix effect [22, 23]. Also for mycotoxins, large differences in SSE have been observed for different varieties of sorghum and rice [10, 24]. However, in multi-mycotoxin analysis, the lot-to-lot variation is often neglected during method validation. According to the US Food and Drug Administration (US-FDA) guidelines, the lot-to-lot variation needs to be evaluated from replicates of at least five different lots of the same matrix [25, 26] for bioanalytical assays and three different lots of the same matrix for mycotoxin assays [27]. In the relevant regulation on mycotoxin determination of the European Union (EU), the matrix-mismatch phenomenon is considered a potential component of uncertainty, but it is not required to validate the assay based on different lots of a matrix [21, 28].

We hypothesise that neglecting the lot-to-lot variation during method validation can lead to an underestimation of *U*_*r*_. The objectives of this study were to (i) apply a practical procedure for the realistic estimation of *U*_*r*_ for measurement results obtained by an LC-MS-based multi-mycotoxin assay and to (ii) determine the contribution of the lot-to-lot variation to *U*_*r*_. This study presents the first calculation of *U*_*r*_ for the determination of mycotoxins in food and feed considering the lot-to-lot variation, and differs significantly from studies which evaluated *U*_*r*_ under repeatability conditions of a single lot of a matrix.

## Materials and methods

### Chemicals and reagents

LC gradient grade methanol and acetonitrile as well as MS grade ammonium acetate and glacial acetic acid (p.a.) were purchased from Sigma-Aldrich (Vienna, Austria). A Purelab Ultra system (ELGA LabWater, Celle, Germany) was used for further purification of reverse osmosis water.

Standards were obtained as gifts either from various research groups or from commercial sources. Stock solutions of each analyte were prepared. Combined working solutions were prepared by mixing the stock solutions of the corresponding analytes for easier handling and were stored at − 20 °C. The multi-analyte standard solution, which contained 66 compounds, was freshly prepared prior to spiking experiments by mixing of the combined working solutions.

### LC-ESI-MS/MS

The measurement method used in this study was described in detail elsewhere [5]. Briefly, the analysis was carried out with a QTrap 5500 MS/MS system (Sciex, Foster City, CA, USA) equipped with a TurboV electrospray ionisation (ESI) source and a 1290 series UHPLC system (Agilent Technologies, Waldbronn, Germany). Chromatographic separation was performed at 25 °C on a Gemini C18-column, 150 × 4.6 mm i.d., 5 μm particle size, equipped with a C18 security guard cartridge, 4 × 3 mm i.d. (both Phenomenex, Torrance, CA, USA). Elution was carried out in binary gradient mode with a flow rate of 1000 μl/min. Both mobile phases contained 5 mM ammonium acetate and were composed of methanol/water/acetic acid 10:89:1 (*v*/*v*/*v*; eluent A) and 97:2:1 (*v*/*v*/*v*; eluent B), respectively. After an initial time of 2 min at 100% A, the proportion of B was increased linearly to 50% within 3 min. Further linear increase of B to 100% within 9 min was followed by a hold time of 4 min at 100% B and 2.5 min column re-equilibration at 100% A. ESI-MS/MS was performed in scheduled multiple reaction monitoring (sMRM) mode both in positive and in negative polarity in two successive chromatographic runs respectively. Confirmation of positive analyte identification is obtained by the acquisition of two sMRM transitions per analyte. The retention time, lowest calibration level, and the spiking concentration for every analyte is given in Table S1 of the Electronic Supplementary Material (ESM).

### Calibration and quantitation

External neat solvent calibration was prepared by dilution of appropriate amounts of multi-analyte standard solution with acetonitrile/water/acetic acid (49.5/49.5/1, *v*/*v*/*v*) to obtain relative concentration levels 1:3:10:30:100:300. To check the linearity of the response, linear, 1/*x* weighted calibration curves were constructed for the neat solvent standards. The construction of calibration curves and peak integration were performed using MultiQuant™2.0.2 software (Sciex, Foster City, CA, USA). Further data evaluation, such as the calculation of the method performance parameter and the associated uncertainties, was carried out in Microsoft Excel 2013 and RStudio Version 1.0.143.

### Representative set of analytes

The described method covers 550 analytes, for which standards with certified purity are available. As representative analytes, 66 fungal metabolites, including all regulated mycotoxins, were chosen. The analytes were evenly distributed over the whole chromatogram (Table S1, ESM) covering both ESI polarities and differences in physiochemical properties (e.g. hydrophobicity, acidity, functional groups). As representative matrices, two matrices from different commodity groups according to [28, 29] were chosen. Figs were taken from the commodity group dried fruits, which are characterised by a high sugar and low water content. Maize was selected as representative matrix of cereal grains, a matrix with high starch and protein and low in water and fat content.

### Spiked samples

An appropriate amount of multi-analyte standard solution (166 μL) was added to 0.25 g of homogenised blank sample. The samples were placed in darkness to avoid analyte degradation (e.g. ergot alkaloids) and stored overnight at room temperature to allow the evaporation of the solvent and to establish an equilibration between analytes and matrix. After this period, the samples were extracted with 1 mL of extraction solvent (acetonitrile/water/acetic acid 79:20:1, *v*/*v*/*v*) and shaken using a rotary shaker (GFL 3017, GFL; Burgwedel, Germany) for 90 min in a horizontal position. In the routine analysis of naturally contaminated material, a higher amount of sample (5 g) is extracted with 20 mL of extraction solvent. As demonstrated before [4, 5, 10, 30], it is sufficient to use only a small amount of blank sample for spiking experiments, which allows for the economical use of mycotoxin standards. The supernatant (300 μL) was transferred into HPLC vials and diluted with the same volume of dilution solvent (acetonitrile/water/acetic acid 20:79:1, *v*/*v*/*v*). After appropriate mixing, 5 μL of the diluted extract was injected into the LC-MS/MS system without further pre-treatment.

### Spiked extracts

Five grams of sample was extracted with 20 mL of extraction solvent. The supernatant was fortified with an appropriate amount of multi-analyte standard, diluted and injected into the LC-MS/MS system as described above.

### Calculation of RE, SSE and RA

The performance characteristics RA, RE and SSE were calculated according to:

$$ {\displaystyle \begin{array}{l} RA=\frac{{\mathrm{area}}_{\mathrm{spiked}\ \mathrm{sample}}}{{\mathrm{area}}_{\mathrm{standard}}}\\ {} RE=\frac{{\mathrm{area}}_{\mathrm{spiked}\ \mathrm{sample}}}{{\mathrm{area}}_{\mathrm{spiked}\ \mathrm{extract}}}\\ {} SSE=\frac{{\mathrm{area}}_{\mathrm{spiked}\ \mathrm{extract}}}{{\mathrm{area}}_{\mathrm{standard}}}\end{array}} $$

The RA, RE and SSE values were calculated as the average of two injections from the same vial.

### Samples

In order to maximise the differences between the individual lots of a matrix, seven lots were collected. For maize, seven lots of different origin and variety (where specified) were obtained as a ground powder from other research groups (Table 1).

**Table 1:**
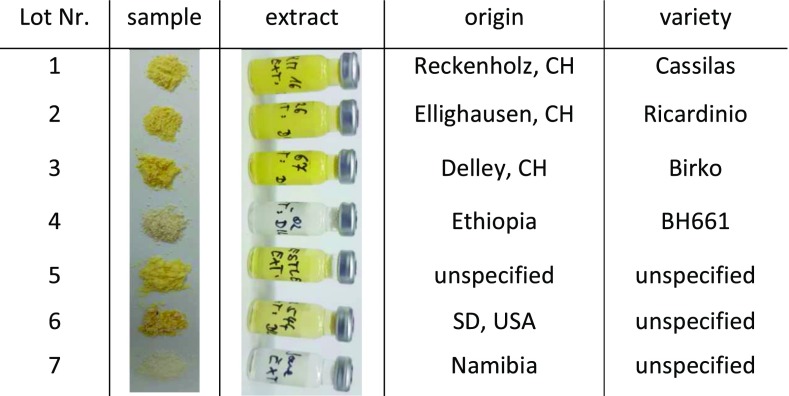
Description of the seven lots of maize used to determine the lot-to-lot variation

Figs were bought in local supermarkets and originated, where specified, from Turkey (Table 2). They differed in specification; however, the variety was not specified. To homogenise the samples, figs where cut in small pieces, frozen in liquid nitrogen and ground using an Osterizer blender (Sunbeam Oster Household Products, Fort Lauderdale, FL, USA).

**Table 2:**
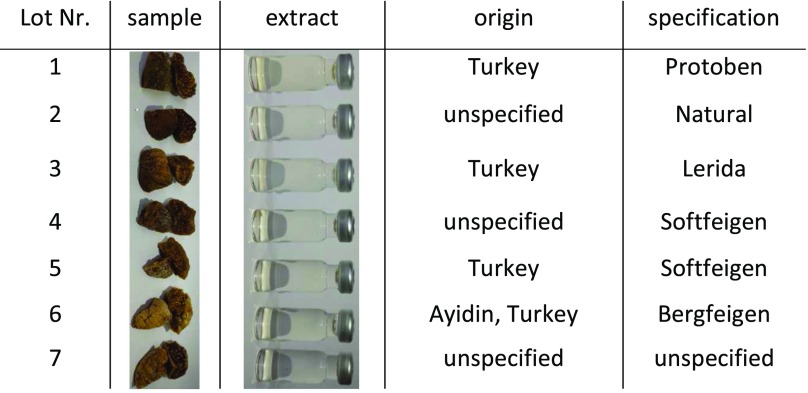
Description of the seven lots of figs used to determine the lot-to-lot variation

## Abbreviations

The following abbreviations (Table 3) were used in the manuscript and were based on the abbreviations used in [31].

**Table 3:** List of abbreviations of the uncertainty components which were used in the manuscript

*U*	Expanded measurement uncertainty close to 95% confidence interval
*U*_*r*_	Relative expanded measurement uncertainty
*U*_*r*, single lot_	Relative expanded measurement uncertainty estimated from intra-laboratory validation data based on a single lot of a matrix
*U*_*r*, lot − to − lot_	Relative expanded measurement uncertainty estimated from intra-laboratory validation data based on seven different lots of a matrix
*U*_*r*, *PT*_	Relative expanded measurement uncertainty estimated from the long-term participation in proficiency test schemes
*u*_*c*_	Combined standard uncertainty
*u*_*r*, *c*_	Relative combined standard uncertainty
*u*_*r*, *i*_	Relative standard uncertainty component
*u*_*r*, *wL*_	Relative standard uncertainty of the within-laboratory precision
*u*_*r*, *RA*_	Relative standard uncertainty of the method bias (apparent recovery, RA)
${u}_{r,{RA}_{\mathrm{single}\ \mathrm{lot}}}$	Relative standard uncertainty of RA based on a single lot of a matrix
${u}_{r,{RA}_{\mathrm{lot}-\mathrm{to}-\mathrm{lot}}}$	Relative standard uncertainty RA based on seven different lots of a matrix
*u*_*r*, *RE*_	Relative standard uncertainty of the recovery of the extraction step (RE)
*u*_*r*, *SSE*_	Relative standard uncertainty of the signal suppression/enhancement (SSE)
*u*_*r*, *X*_	Relative standard uncertainty of the assigned value *X* of the proficiency test scheme
*u*_*r*, *f*_	Maximum relative standard uncertainty based on intra-laboratory validation defined in EC 401/2006 [27]
*RMS*_bias *PT*_	Root mean square of the relative bias values of the result submitted by our laboratory to the assigned value for long-term proficiency test participation

### Method bias

The method bias was estimated by spiking a known amount of the analyte to the homogenised sample aliquots prior to extraction and measuring sample and standard. The method bias was expressed as RA. The relative standard uncertainty of RA (*u*_*r*, *RA*_) was calculated as the relative standard deviation (RSD) of the individual RA values. *u*_*r*, *RA*_ was evaluated from seven aliquots of the same lot (${u}_{r,{RA}_{\mathrm{single}\ \mathrm{lot}}}$) and one aliquot of seven different lots (${u}_{r,{RA}_{\mathrm{lot}-\mathrm{to}-\mathrm{lot}}}$) under repeatability conditions (within one analytical sequence).

### Precision

The within-laboratory reproducibility, also referred to as intermediate precision, was used as an estimate for the random variation of the results generated by applying the method. The relative standard uncertainty of the within-laboratory reproducibility (*u*_*r*, *wL*_) was determined as the RSD of replicate analysis (seven replicates) of the RA value of spiked samples of lot 1. To cover the whole variation that can occur during the sample preparation and measurement, spiked replicates were prepared from aliquots of the same lot directly before the analysis. For the within-laboratory characterisation of reproducibility, the following conditions were used: the same measurement procedure, laboratory and equipment, different operators and repetitions over a long time interval (7 weeks).

### Statement of the result

The measurand is defined as the mass fraction *w* (e.g. μg/kg) and its corresponding expanded measurement uncertainty (*U*) of a mycotoxin in a certain commodity. The range ±*U* around *w* describes the concentration range where the true concentration of the analyte can be found with a probability of approximately 95% (*k* = 2).

$$ w\pm U $$

*w* was calculated by comparing the peak area of the sample to the peak area of the standard in neat solvent and the correction for RA.

$$ w=\frac{{\mathrm{area}}_{\mathrm{sample}}}{{\mathrm{area}}_{\mathrm{standard}}}\times \frac{1}{RA} $$

*U* was calculated from *U*_*r*_:

$$ U=w\times {U}_r $$

*U*_*r*_ was calculated from the relative combined uncertainty (*u*_*r*, *c*_) and the coverage factor (*k*) [31]:

$$ {U}_r=k\times {u}_{r,c}\kern0.5em \mathrm{with}\ \mathrm{k}=2 $$

*u*_*r*,*c*_ was calculated from estimates of precision and trueness according to:

$$ {u}_{r,c}=\sqrt{u_{r, wL}^2+{u}_{r, RA}^2} $$

As individual components of *u*_*r*,*c*_ combine as squares, small contributions can be neglected. The uncertainty associated with the mass concentration of the standard and the LOQ was considered negligible, since commercially available standards with certified purity were spiked at concentration levels far above the LOQ. The contribution of sampling to the uncertainty was not considered since, for mycotoxins, compliance with the maximum limits is established on the basis of the levels determined in the laboratory sample [28]. As *U*_*r*_ was calculated on one concentration level, the assumption that *u*_*r*,*RA*_ and *u*_*r*,*wL*_ do not change significantly over the calibration range was made.

## Determination of the contribution of the lot-to-lot variation to *U*_*r*_

In order to estimate the contribution of the lot-to-lot variation to *U*_*r*_, two different estimates of *U*_*r*_ were calculated. *U*_*r*, single lot_ was calculated based on seven replicates of a single lot of a matrix and does not account for the lot-to-lot variation. *U*_*r*, lot − to − lot_ was calculated based on seven different lots of a matrix and accounts for the lot-to-lot variation.

$$ {\displaystyle \begin{array}{l}{U}_{r,\mathrm{single}\ \mathrm{lot}}=2\times \sqrt{u_{r, wL}^2+{u}_{r,{RA}_{\mathrm{single}\ \mathrm{lot}}}^2}\\ {}{U}_{r,\mathrm{lot}\hbox{-} \mathrm{to}\hbox{-} \mathrm{lot}}=2\times \sqrt{u_{r, wL}^2+{u}_{r,{RA}_{\mathrm{lot}\hbox{-} \mathrm{to}\hbox{-} \mathrm{lot}}}^2}\end{array}} $$

Any increase of *U*_*r*, lot − to − lot_ compared to *U*_*r*, single lot_ was ascribed to the lot-to-lot variation.

## Estimation of *U*_*r*_ from proficiency test results

To establish *U*_*r*_ based on inter-laboratory validation data, the results our laboratory has achieved in proficiency test (PT) schemes, provided by BIPEA, were evaluated. During the last 4 years, 594 results were submitted in 60 PT rounds for aflatoxins, fumonisins, T-2 toxin, HT-2 toxin, deoxynivalenol, nivalenol, ochratoxin and zearalenone in various food and feed matrices. To calculate *U*_*r*_ based on PT results (*U*_*r*, *PT*_), the relative bias value ( bias_*PT*_) of the result submitted by our laboratory (*x*) to the assigned value of the PT (*X*) was calculated according to:

$$ {\mathrm{bias}}_{PT}=\frac{\left(x-X\right)}{X} $$

*U*_*r*, *PT*_ may be estimated from *u*_*r*,*wL*_, the root mean square of the bias_*PT*_ values (*RMS*_bias,*PT*_) and the relative standard uncertainty of *X* (*u*_*r*,*X*_) as described in [15, 29]:

$$ U=k\times \sqrt{u_{r, wL}^2+{RMS}_{\mathrm{bias}, PT}^2+{u}_{r,X}^2} $$

As the bias_*PT*_ values were taken from 60 PT rounds carried out over 4 years, we reasoned that *u*_*r*,*wL*_ was already included in ${RMS}_{\mathrm{bias}, PT}^2$. To avoid double counting of an uncertainty component, *u*_*r*,*wL*_ was not considered a separate contribution in the calculation of *U*_*r*, *PT*_. The average *u*_*r*,*X*_ was 6% and was negligible compared to *RMS*_bias,*PT*_. Therefore, *U*_*r*, *PT*_ was estimated according to:

$$ {U}_{PT}=k\times {RMS}_{\mathrm{bias}, PT}\kern0.5em \mathrm{with}\kern0.5em k=2 $$

## Results

### Contribution of the lot-to-lot variation to *U*_*r*_

In multi-mycotoxin analysis, an uncertainty statement of the measurement result is often missing. Therefore, we employed a practical procedure to estimate *U*_*r*_ for 66 analytes in figs and maize. Multi-mycotoxin methods are commonly validated based on a single lot of a matrix. Neglecting the lot-to-lot variation may lead to an overoptimistic estimate of *U*_*r*_. For the first time, *U*_*r*_ was evaluated based on different lots of a matrix and thus accounts for the lot-to-lot variation.

**Fig. 1:**
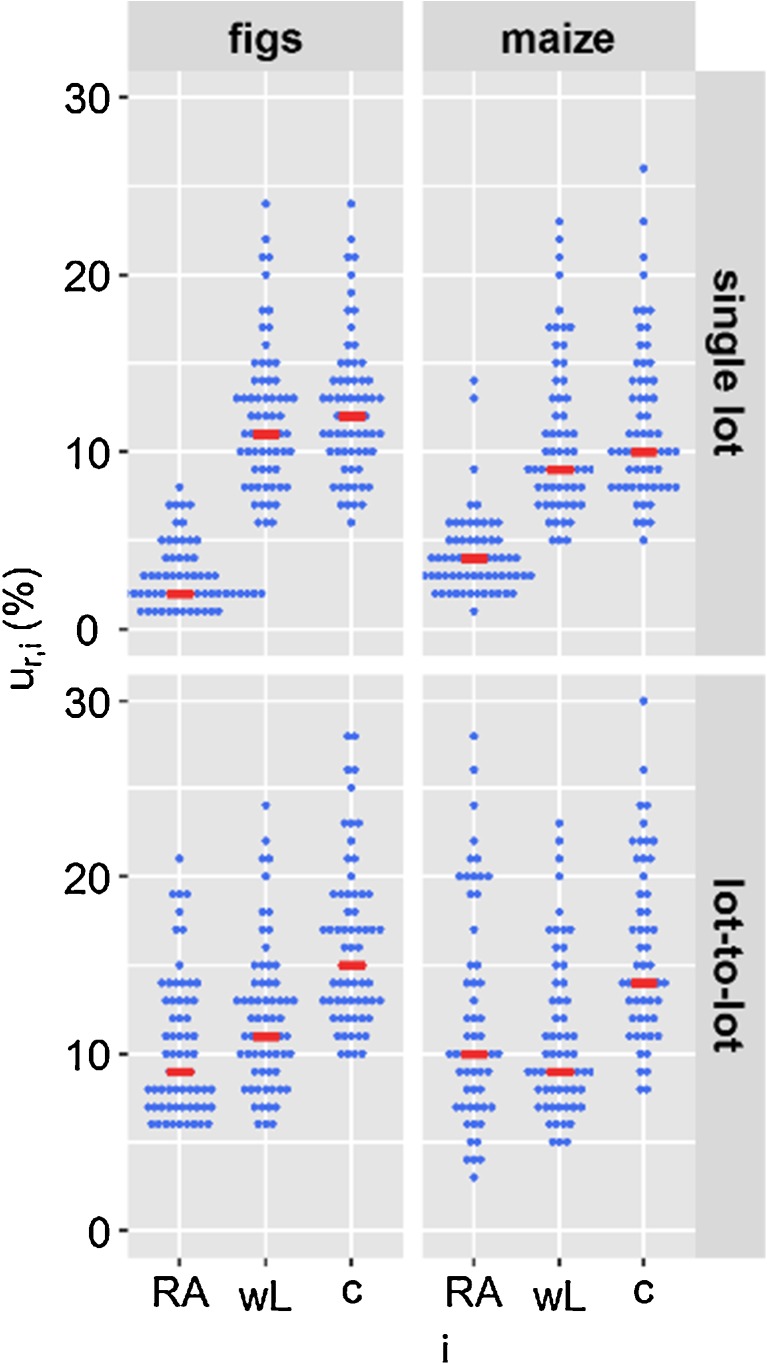
Relative standard uncertainty components (*u*_*r*, *i*_) associated with the method bias RA (*u*_*r*,*RA*_) and precision (*u*_*r,wL*_) which were used to calculate the relative combined standard uncertainty (*u*_*r*, *c*_) for 66 analytes in figs and maize. Top, *u*_*r,RA*_ was evaluated based on a single lot of a matrix; therefore, *u*_*r*, *c*_ does not account for the lot-to-lot variation. Bottom, *u*_*r,RA*_ was evaluated based on seven different lots; thus, *u*_*r*, *c*_ accounts for the lot-to-lot variation. Analytes are represented as blue points, median values are indicated in red

*U*_*r*_ was calculated from the relative combined uncertainty (*u*_*r*, *c*_). *u*_*r*, *c*_ was calculated from estimates of precision (*U*_*r,wL*_) and trueness (*U*_*r,RA*_) (Fig. 1). For the visualisation of the contribution of the lot-to-lot variation to the individual *U*_*r*_ components, the median value of the representative analytes, represented as blue points, was indicated in red. In general, the trend that was observed by comparing the median values of the 66 analytes could also be observed for the individual analytes. The values for the individual analytes are listed in Tables S2 and S3 of the ESM. The median value was only used for illustrative purposes and was not used for the estimation of *U*_*r*_.

**Fig. 2:**
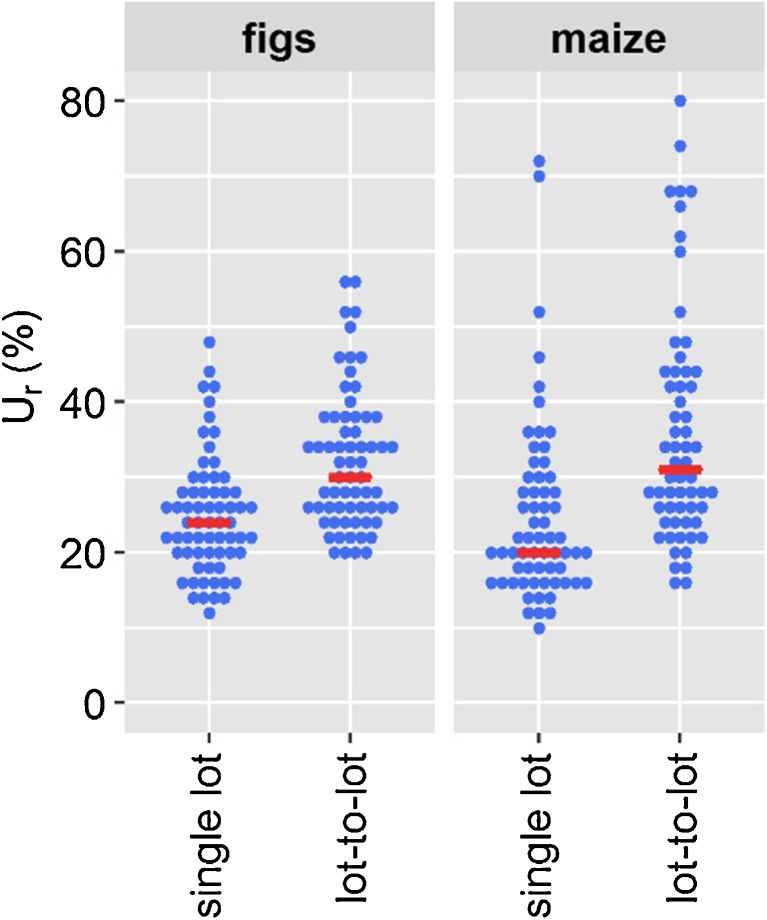
Relative expanded measurement uncertainty (*U*_*r*_) for 66 analytes in figs and maize. *U*_*r*,single lot_ was evaluated from a single lot of a matrix and does not account for the lot-to-lot variation. *U*_*r*,lot-to-lot_ was evaluated based on seven different lots of a matrix and accounts for the lot-to-lot variation. Analytes are represented as blue points; median values are indicated in red

When *u*_*r*,*RA*_ was calculated from replicates of a single lot of a matrix, its contribution to *u*_*r*, *c*_ was negligible (Fig. 1, top). Therefore, *u*_*r*,*wL*_ could be used to estimate *u*_*r*_, if the lot-to-lot variation does not need to be taken into account. When the lot-to-lot variation was taken into account, *u*_*r*,*RA*_ contributed significantly to *u*_*r*, *c*_ (Fig. 1, bottom) which led to an increase in *U*_*r*_. To estimate the contribution of the lot-to-lot variation to *U*_*r*_, *U*_*r*_ evaluated based on seven different lots of a matrix was compared to *U*_*r*_ evaluated based on one lot of a matrix (Fig. 2).

When the lot-to-lot variation was not considered, as is the common practice, the median *U*_*r*_ of the 66 analytes was 24% in figs and 21% in maize. When the lot-to-lot variation was considered, the median *U*_*r*_ of the 66 analytes increased to 31% in both figs and maize. This corresponds to a relative increase of the median *U*_*r*_ of 30% in figs and 50% in maize.

### Contribution of the lot-to-lot variation to the uncertainty of the signal suppression/enhancement and analyte recovery

**Fig. 3:**
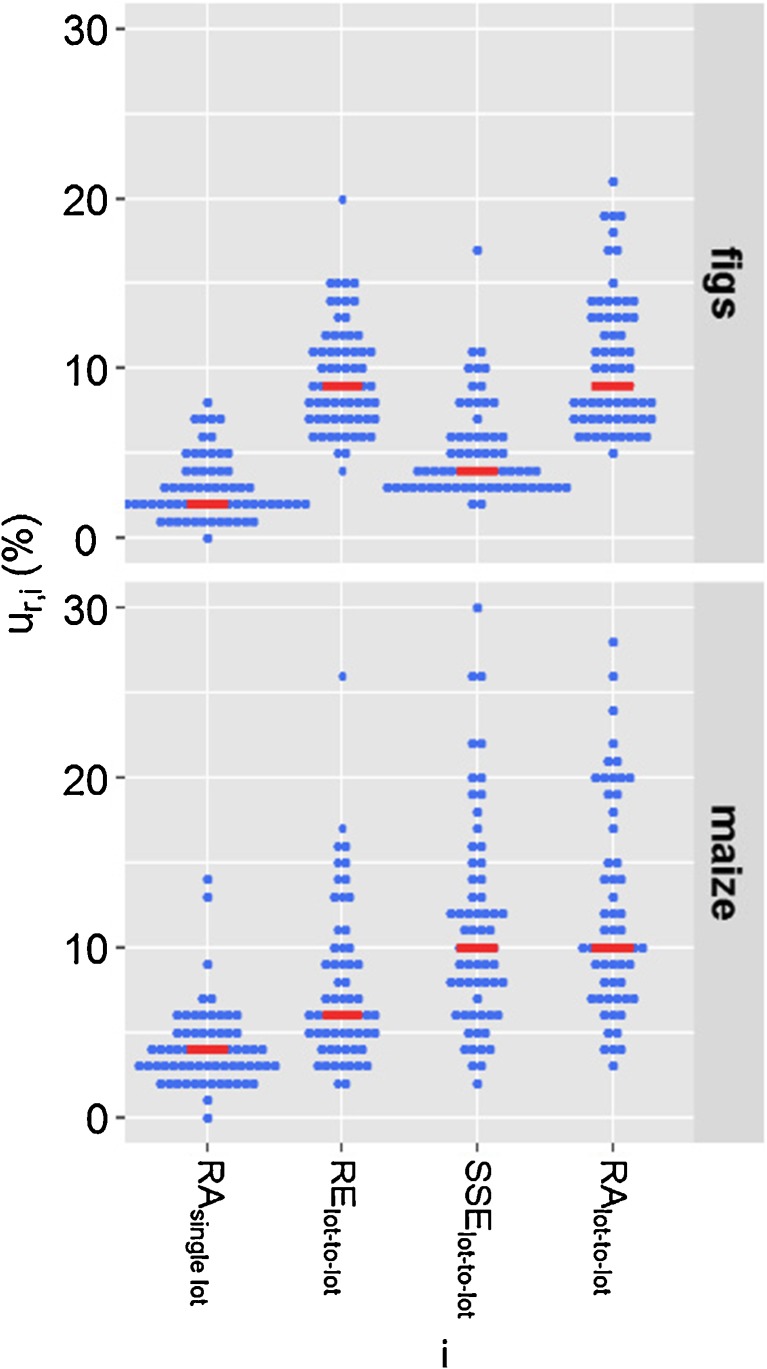
Comparison of the relative standard uncertainty components (*u*_*r*, *i*_) of the method bias RA derived from a single lot of a matrix (*u*_*r*,*RA* single lot_) to the relative standard uncertainty of the analyte recovery (*u*_*r,RE* lot–to lot_), signal suppression/enhancement (*u*_*r,SSE* lot–to lot_) and RA (*u*_*r,RA* lot–to lot_) derived from seven different lots of a matrix. The evaluation was carried out for 66 mycotoxins in figs and maize which are represented as blue points. The median values are indicated in red

Different RA values for individual lots of a matrix contributed to *u*_*r*,*RA*_ and thus *u*_*r*_. The RA value shows the combined effect of RE and SSE. In order to reveal which of the two processes, differences in RE or SSE, contributed to *u*_*r*,*RA*_, we have determined the contribution of the lot-to-lot variation to the relative standard uncertainty of RE (*u*_*r*,*RE*_) and SSE (*u*_*r*,*SSE*_) (Fig. 3). Details can be found in Tables S4 and S5 of the ESM.

*u*_*r,RA* single lot_ was considered to be a measure of the repeatability of the recovery process (spiking, extraction, dilution) and measurement. Therefore, any increase of *u*_*r,RA* lot–to–lot_ compared to *u*_*r,RA* single lot_ would be caused by the lot-to-lot variation. Due to the lot-to-lot variation, the median *u*_*r*,*RA*_ of the 66 analytes increased from 3 to 9% in figs and 4 to 10% in maize. As RA shows the combined effect of RE and SSE, *u*_*r*,*RA*_ can be seen as a combination of *u*_*r*,*RE*_ and *u*_*r*,*SSE*_. In figs, the increase of *u*_*r,RA* lot–to–lot_ compared to *u*_*r,RA* single lot_ was due to an increase of *u*_*r,RE* lot–to–lot_ which was the result of differences in analyte recovery. As *u*_*r,SSE* lot–to–lot_ was similar to *u*_*r,RA* single lot_, relative matrix effects did not contribute to *u*_*r,SSE*_ and *u*_*r,RA*_, respectively. For most compounds in maize, *u*_*r,RE* lot–to–lot_ was similar to *u*_*r,RA* single lot_. For most analytes, differences in analyte recovery were not contributing to *u*_*r*,*RE*_ and *u*_*r*,*RA*_. The increase of *u*_*r,RA* lot–to–lot_ compared to *u*_*r,RA* single lot_ was due to an increase of *u*_*r,SSE* lot–to–lot_ due to differences in SSE. Therefore, for most of the analytes, relative matrix effects contributed to *u*_*r,SSE*_ and *u*_*r*,*RA*_, respectively. In summary, the major contributions of the lot-to-lot variation to *u*_*r*,*RA*_ were differences in analyte recovery in figs and relative matrix effects in maize.

### Measurement uncertainty for the determination of the regulated mycotoxins in figs and maize

**Fig. 4:**
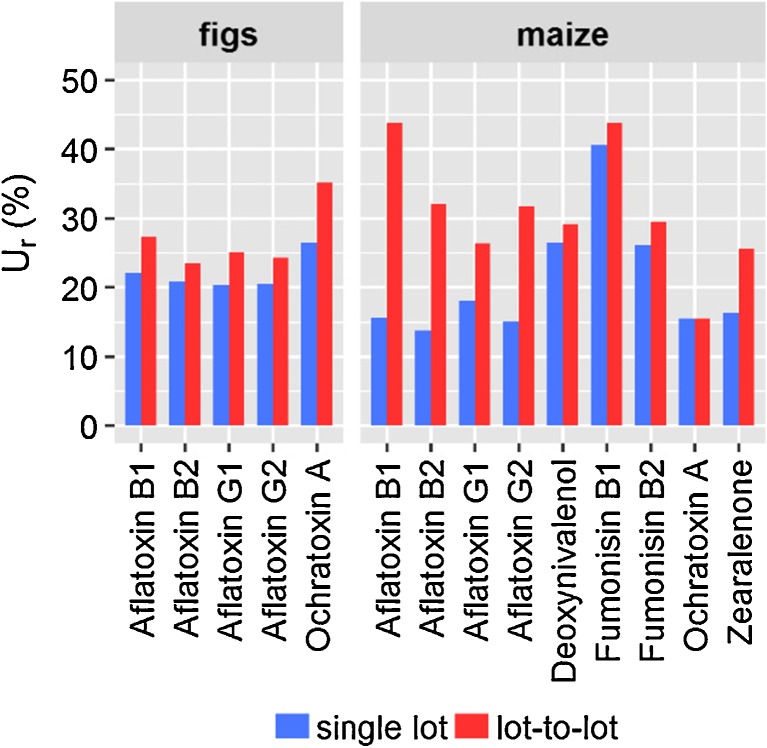
Comparison of the relative expanded measurement uncertainty neglecting the lot-to-lot variation (*U*_*r*,single lot_) and accounting for the lot-to-lot variation (*U*_*r*,lot-to-lot_) for the regulated mycotoxins in figs and maize

To visualise the contribution of lot-to-lot variation to *U*_*r*_ for the regulated mycotoxins in figs and maize, *u*_*r,*single lot_ was compared to *u*_*r,*lot–to–lot_ (Fig. 4).

The trend which was observed by the comparison of the median values of the representative sets of analytes was also observed for the individual regulated mycotoxins in both matrices. *U*_*r*_ increased due to the lot-to-lot variation. For the aflatoxins in maize, the contribution was especially high and could be ascribed to relative matrix effects.

### Estimation of the measurement uncertainty based on the results of PT schemes

**Fig. 5:**
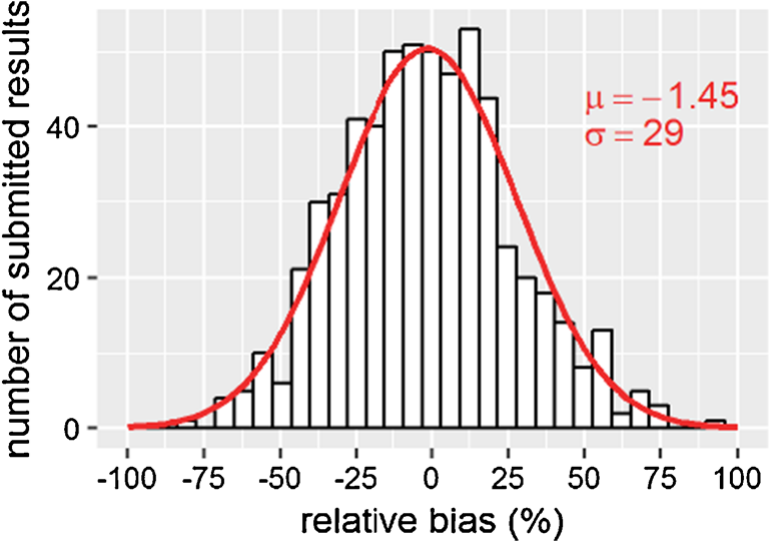
Histogram of the relative bias values of the results submitted by our laboratory to the assigned value of the proficiency test scheme achieved from 2013 to 2017 for aflatoxins, fumonisins, T2, HT2, deoxynivalenol, nivalenol, ochratoxin and zearalenone in various food and feed matrices. A normal distribution curve with mean μ and standard deviation σ is fitted to the histogram in red

The spread of the bias_*PT*_ values, which describe the deviation of the submitted result to the assigned value, was used to estimate *U*_*r*, *PT*_. Upon visual inspection, the bias_*PT*_ values were unbiased and normally distributed (Fig. 5).

For unbiased results, the RMS equals the standard deviation of the bias_*PT*_ values which was determined with 29% and corresponds to *U*_*r*, *PT*_ = 58%. From 2013 to 2017, 95% of the submitted concentration values were within ± 58% of the assigned concentration.

## Discussion

### Choosing an adequate procedure for the calculation of the uncertainty budget of a multi-mycotoxin method

*U*_*r*_ for the multi-mycotoxin method was calculated from intra-laboratory validation data for 66 analytes in figs and maize and from the participation in PT schemes for the regulated mycotoxins (except patulin) and nivalenol, T-2 toxin and HT-2 toxin in various food and feed matrices. The following paragraph lists common approaches for the calculation of *U*_*r*_ and aims to discuss their applicability to calculate *U*_*r*_ for a multi-mycotoxin method.

The “bottom-up” approach: The measurement uncertainty can be calculated based on the identification, quantification and combination of all individual components of measurement uncertainty (i.e. bottom-up approach). A rigorous bottom-up approach, as it is proposed in the “Guide to the Expression of Uncertainty in Measurement” [12], is considered impractical for multi-analyte methods, covering several hundred substances, as the calculation of the individual uncertainty components for each analyte-matrix combination is too laborious [15, 32]. Furthermore, effects which were not considered as potential error sources may lead to an underestimation of the measurement uncertainty.

The “top-down” approach: By a top-down approach, as proposed by ISO\TS 21748 [16], the performance of a well-defined measurement method is evaluated by an inter-laboratory comparison (ILC) study. The reproducibility standard deviation (*RSD*_*R*_) is then used to calculate *U*_*r*_. ILC studies are only available for methods covering a limited number of analytes in a few matrices [33, 34]. No inter-laboratory comparison studies or certified reference materials (CRM) are available that cover the scope of the described multi-mycotoxin assay. Incomplete variation of certain influences (e.g. lot-to-lot variation) in the highly homogenous CRMs may lead to an underestimation of the measurement uncertainty.

Proficiency tests: *U*_*r*_ may also be estimated from results achieved during the participation in PT schemes. In contrast to ILC studies, laboratories can employ their own test method. Although in multi-residue analysis different methods (GC-MS and LC-MS) were used, it has been verified that the performance of the methods in the PTs does not depend strongly on the extraction method or the detection techniques [35]. Therefore, the RSD of the PT was used to estimate *U*_*r*_ of the individual methods which successfully participated in PT schemes. The mean RSD of EU-based PT studies for pesticides in fruit and vegetables has ranged at approximately 25% [35, 36]. Therefore the EU member states have adopted a default value of *U*_*r*_ = 50% for pesticide residues in food consignment entering the EU [15].

The performance of the presented method has been evaluated by the participation in PT schemes provided by BIPEA. The methods used in PTs for mycotoxins included LC-MS, GC-MS, LC-UV, LC-FLD and ELISA. As it is unsure whether the individual methods perform similar in PTs, the RSD value of the PT scheme cannot be used for the estimation of *U*_*r*_. Therefore, *U*_*r*, *PT*_ was calculated from the spread of submitted results of the laboratory to the assigned values. As the raw material and the concentration of the analyte change for every PT round, the influence of the lot-to-lot variation and different concentration ranges were considered as potential uncertainty components. *U*_*r*, *PT*_ for regulated mycotoxins in food and feed was estimated with 58% similar to the average *U*_*r*_ of about 50% achieved in pesticide analysis [15, 35]. *U*_*r*, *PT*_ might overestimate *U*_*r*_ since the criteria for the homogeneity of the samples are not as strict as for CRMs.

Intra-laboratory validation: Intra-laboratory validation can be carried out by estimating the uncertainty of precision and trueness based on measurements of spiked samples [14, 17, 21, 31]. *U*_*r*_ was calculated for a representative set of analyte/matrix combinations and not for each individual analyte/matrix combination as recommended for multi-residue analysis [15]. We considered *U*_*r*_, determined for a representative set of analyte and matrices, a valid estimator for *U*_*r*_ for all analytes included in the assay. Due to the limited availability of CRMs of mycotoxins in food and feed, we evaluated *u*_*r*,*wL*_ and *u*_*r*,*RA*_ using spiked samples. A similar approach has been used previously for multi-mycotoxin methods in sorghum and feed where *U*_*r*_ was calculated from *u*_*r*,*wL*_ (measurements were carried out at 3 different days) and *u*_*r*,*RA*_ (from replicates of the same lot of a matrix) [24, 37]. Our approach differed in calculating *u*_*r*,*RA*_ from seven different lots of the matrix and thus accounting for the lot-to-lot variation. Furthermore, we calculated *u*_*r*,*wL*_ from one replicate at 7 different days distributed over a longer time period (7 weeks). This should provide a more realistic measure of *u*_*r*,*wL*_ and *u*_*r*,*RA*_.

### Method performance in regard to the official EC decisions and regulations on mycotoxin determination

Performance criteria for compliance testing according to European Commission Decision EC 1881/2006 [2] are set in European Commission Decision EC 401/2006 [28]. Methods which cannot be validated by ILC studies, like the described multi-mycotoxin assay, may be validated by in-house studies. For the analytes under investigation, the achieved *u*_*r*, *c*_ needs to comply with the maximum standard uncertainty (*k* = 1) defined by the fitness-for-purpose function (*u*_*f*_). The maximum relative standard uncertainty *u*_*r*, *f*_ converges to approximately 20% at concentration levels below 500 μg/kg, which corresponds to a maximum *U*_*r*_ of 40%. As the EU legislation requires that *U*_*r*_ is evaluated based on a single lot, compliance to this criterion is achieved by *U*_*r*, single lot_ < 40%. All regulated mycotoxin-matrix combinations showed acceptable *U*_*r*_ values. Therefore, we see the method suitable for compliance testing according to European Commission Decision EC 1881/2006.

### Fit-for-purpose *U* for a LC-MS-based multi-mycotoxin method

For LC-MS-based multi-mycotoxin determination, we propose one fit-for-purpose *U*_*r*_ for all analyte/matrix combinations independent of the concentration level of the analyte. In our view, a fit-for-purpose *U*_*r*_ should provide maximum utility by minimising the effort in validation and at the same time providing a realistic estimation for *U*_*r*_. Similar to Stroka and Maragos [38], we favour a fixed over a concentration-dependent *U*_*r*_ as all analytes were subjected to the same measurement principle. During intra-laboratory validation, we found that the main contribution factors to *U*_*r*_ were the within-laboratory precision and the lot-to-lot variation, which we consider to be independent of the concentration of the analyte. For the described multi-mycotoxin assay, we propose a fit-for-purpose *U*_*r*_ of 50%. It applies to the measurement procedure (i.e. extraction, dilution and mass spectrometric determination) for analytes where a standard with certified purity was available and does not include uncertainty components arising from sampling or concentration levels close to the limit of quantitation. Intra-laboratory validation showed that respectively 90 and 80% of the 66 representative analytes in figs and maize had an associated *U*_*r*, lot − to − lot_ < 50%. *U*_*r*_ derived from long-term participation in PT schemes for regulated mycotoxins in various food and feed matrices was estimated with 58%. The proposed fit-for-purpose *U*_*r*_ of 50% is also in line with the default value of *U*_*r*_ = 50% that was set for LC-MS-based multi-residue analysis, which depends on the same detection principle and covers analytes in the same concentration range as LC-MS-based multi-mycotoxin analysis. Clearly, this approach cannot replace method validation. The latter is necessary to prove that the method fulfils other requirements (e.g. recovery, matrix effects), pointed out in EC 401/2006. Only if those requirements are passed, a default *U*_*r*_ of 50% might be assumed.

## Conclusion

This study presents the first calculation of the relative expanded measurement uncertainty (*U*_*r*_) of measurement results obtained by an LC-MS-based multi-mycotoxin assay in food and feed matrices accounting for the lot-to-lot variation. We have shown that neglecting the lot-to-lot variation during method validation can lead to an underestimation of *U*_*r*_. We applied a straightforward procedure for the estimation of *U*_*r*_ and determined the contribution of the lot-to-lot variation to *U*_*r*_. *U*_*r*_ was estimated based on intra-laboratory validation data for a representative set of analytes from the uncertainty of the within-laboratory precision, *u*_*r*,*wL*_, (seven replicates of the same lot of a matrix distributed over a time frame of seven weeks) and the uncertainty of the method bias, *u*_*r*,*RA*_, (seven replicates measured under repeatability conditions). The contribution of the lot-to-lot variation to *U*_*r*_ was estimated by taking the replicates for the determination of *u*_*r*,*RA*_ from seven different lots instead of single lot a matrix as is the common practice. The lot-to-lot variation contributed to *u*_*r*,*RA*_ which resulted in an increase of *U*_*r*_ for most analytes in figs and maize. The major contributions of the lot-to-lot variation to *u*_*r*,*RA*_ were differences in analyte recovery in figs and relative matrix effects in maize. *U*_*r*_ was also estimated from the long-term participation in PT schemes with 58%. Accounting for the lot-to-lot variation leads to a more realistic estimate of *U*_*r*_ and should be required by the official guidelines on mycotoxin determination. Provided proper validation, a fit-for-purpose *U*_*r*_ of 50% was proposed for measurement results obtained by a LC-MS/MS-based multi-mycotoxin assay, independent of the concentration of the analytes.

## Electronic supplementary material

ESM 1 (PDF 706 kb)
