# Supplementary material for: The contribution of lot-to-lot variation to the measurement uncertainty of an LC-MS-based multi-mycotoxin assay
Source: Anal Bioanal Chem. 2018 May 1;410(18):4409–18. doi: 10.1007/s00216-018-1096-5 (PMC6021480; doi:10.1007/s00216-018-1096-5)
Supplement: Supplementary file 1 — (PDF 706 kb) [file 216_2018_1096_MOESM1_ESM.pdf]

Analytical and Bioanalytical Chemistry

Electronic Supplementary Material

The contribution of lot-to-lot variation to the measurement uncertainty of an LC-MS based multi-mycotoxin assay

David Stadler, Michael Sulyok, Rainer Schuhmacher, Franz Berthiller, Rudolf Krska

Table S1

Table 1: Retention time (t_R), lowest calibration level (LCL) and spiking concentration (c_{spiked}).

analyte	t_R (min)	LCL (µg/kg)	c_{inj} (µg/kg)	c_{spiked} (µg/kg)
15-Acetyldeoxynivalenol	7.1	1.9	65	520
15-Hydroxyculmorin	9.9	2.1	6.9	56
Aflatoxin B1	8.4	0.05	4.7	38
Aflatoxin B2	8.1	0.05	4.7	38
Aflatoxin G2	7.5	0.14	4.7	38
Aflatoxin G1	7.8	0.14	4.7	38
Aflatoxin M1	7.5	0.05	4.7	38
Brevianamid F	7.2	0.20	6.8	55
Cereulide	15.7	0.19	19	150
Cyclopenin	8.2	0.08	8.1	65
Cytochalasin J	9.4	0.61	20	162
Destruxin A	10.3	0.07	6.7	54
Diacetoxyscirpenol	8.2	0.14	14	114
Enniatin B	13.6	0.002	0.2	1
Ergocristinine	9.4	0.06	0.6	5
Ergocristine	8.2	0.08	0.8	7
Ergotaminin	7.4	0.02	0.8	7
Ergotamine	7.6	0.08	2.7	22
Fumagillin	12.5	2.9	10	77
Fumigaclavine C	6.9	0.29	29	229
Fumonisin B1	9.3	1.7	168	1341
Fumonisin B2	11.1	1.7	169	1352
Fumonisin B3	10.2	1.7	168	1344
Gliotoxin	8.5	0.35	35	279
Griseofulvin	9.5	0.17	17	139
Herquline A	5.4	0.02	2.1	17
HT-2 toxin	9.4	1.4	14	114
Kojic acid	3.0	6.4	212	1697
Monoacetoxyscirpenol	7.5	1.4	14	111
Mycophenolic acid	10.2	0.63	21	167
Neosolaniol	6.4	0.55	55	436
Ochratoxin A	11.7	0.27	27	218
O-Methylsterigmatocystin	10.6	0.10	10	84
Penitrem A	13.3	0.11	11	89
Phomopsin A	7.2	0.81	81	646
Questiomycin A	8.6	0.88	8.8	71
Quinolactacin A	7.3	0.03	3.4	27
Roquefortine C	8.6	6.1	20	162
Secalonic acid D	12.9	0.87	87	698
Sterigmatocystin	11.9	0.07	7.4	60
T-2 toxin	10.3	0.42	14	113
T2-Triol	8.6	2.2	22	173
3-Acetyldeoxynivalenol	7.1	1.4	14	114
3-Nitropropionic acid	2.8	0.59	59	471
alpha-Zearalenol	11.4	0.14	14	114
Alternariol	10.6	0.07	7.4	60
Alternariolmethylether	12.4	0.14	14	115
Altersetin	15.0	0.84	8.4	67
Andrastin A	12.6	0.08	8.4	67
Apicidin	12.4	0.15	15	123
Asperphenamate	12.6	0.03	0.8	7
Averufin	15.6	0.08	8.4	67
Butenolid	2.6	0.66	66	524
Citrinin	11.3	0.22	22	179
Deoxynivalenol	5.8	0.14	14	110
Equisetin	14.9	0.07	7.1	57
Fumiquinazolin A	10.1	0.08	7.7	61
Fusarenon-X	6.3	1.4	14	110
Lotaustralin	5.2	1.5	150	1197
Malformin A	11.5	0.07	6.7	54
Meleagrin	7.5	0.24	24	189
Moniliformin	3.2	0.35	35	281
Nivalenol	4.7	1.4	143	1143
Norsolorinic acid	16.5	0.13	13	108
Patulin	4.8	0.63	21	169
Pseurotin A	8.2	0.49	49	393
Zearalenone-sulfate	11.0	0.15	15	116
Zearalenone	11.5	0.14	14	114

Table S2

Table 2: Calculation of the uncertainty budget for 66 mycotoxins in figs.

accounting for the lot-to-lot variation

analyte	$u_{r,RA}$ (%)	$u_{r,wL}$ (%)	$u_{r,c}$ (%)	$U_{c(k=2)}$ (%)
15-Acetyldeoxynivalenol	7	11	13	26
15-Hydroxyculmorin	8	17	19	38
Aflatoxin B1	8	11	14	27
Aflatoxin B2	6	10	12	24
Aflatoxin G2	7	10	12	24
Aflatoxin G1	8	10	13	25
Aflatoxin M1	6	12	13	27
Brevianamid F	6	12	13	27
Cereulide	14	24	28	56
Cyclopenin	7	13	14	29
Cytochalasin J	9	10	13	26
Destruxin A	8	7	11	22
Diacetoxyscirpenol	6	10	12	24
Enniatin B				n.e.
Ergocristinine	13	10	17	33
Ergocristine	10	8	13	26
Ergotaminin				n.e.
Ergotamine	14	9	16	32
Fumagillin	13	9	16	32
Fumigaclavine C	7	7	11	21
Fumonisin B1	9	15	17	34
Fumonisin B2	10	16	19	38
Fumonisin B3	10	17	19	39
Gliotoxin	8	12	15	30
Griseofulvin	8	9	12	24
Herquline A	7	14	15	31
HT-2 toxin	6	8	10	21
Kojic acid				n.e.
Monoacetoxyscirpenol	10	13	17	34
Mycophenolic acid	14	13	19	39
Neosolaniol	8	8	11	23
Ochratoxin A	12	13	18	35
O-Methylsterigmatocystin	8	12	14	28
Penitrem A	14	22	26	52
Phomopsin A	8	18	20	40
Questiomycin A	7	15	17	34
Quinolactacin A	8	11	14	27
Roquefortine C	11	7	13	26
Secalonic acid D	12	14	19	38
Sterigmatocystin	13	11	17	34
T-2 toxin	9	9	13	26
T2-Triol	21	11	23	47
3-Acetyldeoxynivalenol	7	10	13	25
3-Nitropropionic acid	11	13	17	34
alpha-Zearalenol	12	7	14	28
Alternariol	10	6	12	24
Alternariolmethylether	11	6	12	25
Altersetin	19	21	28	56
Andrastin A	14	15	21	42
Apicidin	15	21	25	51
Asperphenamate	18	14	23	46
Averufin	19	11	22	44
Butenolid	6	18	19	39
Citrinin	19	13	23	47
Deoxynivalenol	7	8	10	21
Equisetin	17	20	26	52
Fumiquinazolin A	11	13	17	34
Fusarenon-X	7	8	10	21
Lotaustralin	6	10	11	23
Malformin C	9	6	11	21
Meleagrin	14	11	17	35
Moniliformin	7	15	16	33
Nivalenol	6	8	10	21
Norsolorinic acid	17	13	21	43
Patulin	6	13	14	29
Pseurotin A	11	14	18	36
Zearalenone-sulfate	13	12	17	35
Zearalenone	13	8	15	31

not accounting for the lot-to-lot variation

analyte	$u_{r,RA}$ (%)	$u_{r,wL}$ (%)	$u_{r,c}$ (%)	$U_{r,(k=2)}$ (%)
15-Acetyldeoxynivalenol	6	11	13	25
15-Hydroxyculmorin	5	17	18	35
Aflatoxin B1	1	11	11	22
Aflatoxin B2	3	10	10	21
Aflatoxin G2	2	10	10	20
Aflatoxin G1	2	10	10	20
Aflatoxin M1	2	12	12	24
Brevianamid F	3	12	12	25
Cereulide	4	24	24	49
Cyclopenin	2	13	13	26
Cytochalasin J	4	10	11	22
Destruxin A	1	7	7	14
Diacetoxyscirpenol	2	10	10	21
Enniatin B				n.e.
Ergocristinine	5	10	11	22
Ergocristine	7	8	11	23
Ergotaminin				n.e.
Ergotamine	5	9	10	20
Fumagillin	6	9	11	22
Fumigaclavine C	2	7	8	15
Fumonisin B1	3	15	15	30
Fumonisin B2	2	16	16	32
Fumonisin B3	2	17	17	34
Gliotoxin	3	12	13	26
Griseofulvin	2	9	9	18
Herquline A	1	14	14	27
HT-2 toxin	2	8	9	18
Kojic acid				n.e.
Monoacetoxyscirpenol	5	13	14	28
Mycophenolic acid	5	13	14	29
Neosolaniol	1	8	8	17
Ochratoxin A	3	13	13	27
O-Methylsterigmatocystin	2	12	12	24
Penitrem A	2	22	22	44
Phomopsin A	7	18	19	38
Questiomycin A	7	15	16	33
Quinolactacin A	2	11	11	23
Roquefortine C	7	7	10	19
Secalonic acid D	2	14	14	29
Sterigmatocystin	1	11	11	22
T-2 toxin	1	9	9	19
T2-Triol	8	11	13	27
3-Acetyldeoxynivalenol	5	10	11	23
3-Nitropropionic acid	2	13	13	26
alpha-Zearalenol	2	7	7	14
Alternariol	4	6	7	14
Alternariolmethylether	2	6	6	12
Altersetin	2	21	21	42
Andrastin A	2	15	15	31
Apicidin	3	21	21	42
Asperphenamate	4	14	15	29
Averufin	2	11	11	23
Butenolid	1	18	18	37
Citrinin	1	13	13	27
Deoxynivalenol	2	8	8	16
Equisetin	1	20	20	40
Fumiquinazolin A	3	13	13	26
Fusarenon-X	3	8	8	16
Lotaustralin	1	10	10	20
Malformin C	3	6	7	13
Meleagrin	4	11	12	23
Moniliformin	1	15	15	30
Nivalenol	2	8	8	17
Norsolorinic acid	1	13	13	26
Patulin	3	13	14	27
Pseurotin A	3	14	14	29
Zearalenone-sulfate	2	12	12	24
Zearalenone	2	8	8	16

n.e.: not evaluated

Table 3: Calculation of the uncertainty budget for 66 mycotoxins in maize.

Table S3

accounting for the lot-to-lot variation

analyte	$u_{r,RA}$ (%)	$u_{r,wL}$ (%)	$u_{r,c}$ (%)	$U_{r,(k=2)}$ (%)
15-Acetyldeoxynivalenol	4	9	10	20
15-Hydroxyculmorin	24	23	34	67
Aflatoxin B1	21	7	22	44
Aflatoxin B2	15	7	16	32
Aflatoxin G2	14	7	16	32
Aflatoxin G1	10	8	13	26
Aflatoxin M1	7	7	10	20
Brevianamid F	9	8	12	24
Cereulide	53	33	62	124
Cyclopenin	6	6	8	17
Cytochalasin J	9	6	11	22
Destruxin A	12	5	13	26
Diacetoxyscirpenol	14	5	14	29
Enniatin B				n.e.
Ergocristinine	35	11	37	74
Ergocristine	20	12	24	47
Ergotaminin				n.e.
Ergotamine	20	17	26	52
Fumagillin	21	11	24	47
Fumigaclavine C	8	8	11	23
Fumonisin B1	9	20	22	44
Fumonisin B2	7	13	15	30
Fumonisin B3	9	17	19	39
Gliotoxin	22	36	42	84
Griseofulvin	12	6	13	26
Herquline A	7	10	12	25
HT-2 toxin	10	9	14	27
Kojic acid	4	17	17	35
Monoacetoxyscirpenol	10	10	14	28
Mycophenolic acid	6	9	11	21
Neosolaniol	32	10	34	67
Ochratoxin A	3	7	8	16
O-Methylsterigmatocystin	20	8	21	42
Penitrem A	14	15	21	41
Phomopsin A	15	13	20	40
Questiomycin A	10	6	12	24
Quinolactacin A	7	5	9	18
Roquefortine C				n.e.
Secalonic acid D	12	9	15	30
Sterigmatocystin	7	9	11	22
T-2 toxin	5	8	9	19
T2-Triol	20	22	30	59
3-Acetyldeoxynivalenol	13	13	18	36
3-Nitropropionic acid	19	11	22	44
alpha-Zearalenol	20	7	21	42
Alternariol	47	9	48	96
Alternariolmethylether	11	7	13	27
Altersetin	5	16	17	33
Andrastin A	39	11	40	80
Apicidin	8	9	12	23
Asperphenamate	17	9	19	38
Averufin	10	14	18	35
Butenolid	28	17	33	66
Citrinin	51	60	79	158
Deoxynivalenol	9	12	15	29
Equisetin	19	11	22	44
Fumiquinazolin A	10	9	14	27
Fusarenon-X	7	8	11	22
Lotaustralin	6	16	17	33
Malformin C	4	14	14	28
Meleagrin	8	15	17	33
Moniliformin	26	18	31	63
Nivalenol	33	10	34	68
Norsolorinic acid	11	9	14	28
Patulin				n.e.
Pseurotin A	11	21	23	47
Zearalenone-sulfate	10	10	14	27
Zearalenone	10	8	13	26

not accounting for the lot-to-lot variation

analyte	$u_{r,RA}$ (%)	$u_{r,wL}$ (%)	$u_{r,c}$ (%)	$U_{r,(k=2)}$ (%)
15-Acetyldeoxynivalenol	6	9	11	23
15-Hydroxyculmorin	5	23	23	47
Aflatoxin B1	3	7	8	16
Aflatoxin B2	2	7	7	14
Aflatoxin G2	4	7	8	15
Aflatoxin G1	4	8	9	18
Aflatoxin M1	3	7	8	16
Brevianamid F	4	8	9	18
Cereulide	13	33	35	71
Cyclopenin	3	6	7	14
Cytochalasin J	4	6	7	14
Destruxin A	3	5	6	12
Diacetoxyscirpenol	2	5	5	10
Enniatin B				n.e.
Ergocristinine	9	11	14	28
Ergocristine	7	12	14	28
Ergotaminin				n.e.
Ergotamine	6	17	18	36
Fumagillin	7	11	13	26
Fumigaclavine C	4	8	9	18
Fumonisin B1	4	20	20	41
Fumonisin B2	2	13	13	26
Fumonisin B3	1	17	17	34
Gliotoxin	4	36	36	72
Griseofulvin	2	6	6	12
Herquline A	3	10	10	21
HT-2 toxin	6	9	11	21
Kojic acid	5	17	18	36
Monoacetoxyscirpenol	6	10	12	23
Mycophenolic acid	3	9	10	19
Neosolaniol	2	10	10	20
Ochratoxin A	3	7	8	15
O-Methylsterigmatocystin	2	8	8	17
Penitrem A	3	15	15	31
Phomopsin A	6	13	14	29
Questiomycin A	5	6	8	15
Quinolactacin A	3	5	6	11
Roquefortine C				n.e.
Secalonic acid D	3	9	9	19
Sterigmatocystin	4	9	10	19
T-2 toxin	3	8	8	17
T2-Triol	14	22	26	52
3-Acetyldeoxynivalenol	2	13	13	27
3-Nitropropionic acid	3	11	11	23
alpha-Zearalenol	4	7	8	17
Alternariol	3	9	10	20
Alternariolmethylether	4	7	8	16
Altersetin	2	16	16	32
Andrastin A	3	11	11	22
Apicidin	5	9	10	20
Asperphenamate	5	9	10	20
Averufin	3	14	15	30
Butenolid	5	17	17	35
Citrinin	6	60	60	121
Deoxynivalenol	6	12	13	27
Equisetin	3	11	12	23
Fumiquinazolin A	4	9	10	21
Fusarenon-X	4	8	9	19
Lotaustralin	2	16	16	31
Malformin C	2	14	14	27
Meleagrin	5	15	15	31
Moniliformin	6	18	18	37
Nivalenol	2	10	11	21
Norsolorinic acid	5	9	10	20
Patulin				n.e.
Pseurotin A	4	21	21	42
Zearalenone-sulfate	3	10	10	20
Zearalenone	2	8	8	16

n.e.: not evaluated

Table S4

Table 4: Evaluation of the performance parameter RE, SSE ,RA and their associated relative standard uncertainties ($u_{r,RE}$, $u_{r,SSE}$, $u_{r,RA}$) for 66 mycotoxins in figs.

1 aliquot of 7 different lots analysed under repeatability conditions

analyte	RE (%)	$u_{r,RE}$ (%)	SSE (%)	$u_{r,SSE}$ (%)	RA (%)	$u_{r,RA}$ (%)
15-Acetyldeoxynivalenol	92	10	85	3	78	7
15-Hydroxyculmorin	91	8	104	3	94	8
Aflatoxin B1	90	7	84	3	76	8
Aflatoxin B2	90	7	85	4	76	6
Aflatoxin G2	92	7	88	3	81	7
Aflatoxin G1	88	5	95	5	84	8
Aflatoxin M1	88	6	96	4	85	6
Brevianamid F	87	7	86	3	75	6
Cereulide	86	12	97	3	84	14
Cyclopenin	91	8	104	3	95	7
Cytochalasin J	88	9	101	4	89	9
Destruxin A	91	8	90	4	82	8
Diacetoxyscirpenol	92	7	95	3	87	6
Enniatin B	85	12	95	3	80	13
Ergocristinine	91	9	79	8	72	13
Ergocristine	84	8	95	3	80	10
Ergotaminin	78	7	86	6	66	5
Ergotamine	109	13	64	8	70	14
Fumagillin	85	9	96	8	81	13
Fumigaclavine C	94	7	88	3	83	7
Fumonisin B1	56	10	94	3	53	9
Fumonisin B2	71	10	102	3	73	10
Fumonisin B3	70	8	132	2	93	10
Gliotoxin	80	9	100	3	80	8
Griseofulvin	90	6	96	3	87	8
Herquline A	79	11	96	5	76	7
HT-2 toxin	97	11	85	11	82	6
Kojic acid	n.e.		n.e.		n.e.	
Monoacetoxyscirpenol	92	12	93	4	85	10
Mycophenolic acid	88	12	105	6	93	14
Neosolaniol	88	9	101	5	89	8
Ochratoxin A	84	11	97	3	83	12
O-Methylsterigmatocystin	87	6	94	4	82	8
Penitrem A	71	11	286	4	203	14
Phomopsin A	67	9	88	10	59	8
Questiomycin A	94	7	101	2	95	7
Quinolactacin A	83	9	99	3	82	8
Roquefortine C	80	11	86	6	69	11
Secalonic acid D	92	11	119	7	109	12
Sterigmatocystin	82	10	100	4	82	13
T-2 toxin	88	8	97	4	85	9
T2-Triol	89	20	104	17	89	21
3-Acetyldeoxynivalenol	88	10	56	9	49	7
3-Nitropropionic acid	84	6	58	10	49	11
alpha-Zearalenol	93	8	88	5	82	12
Alternariol	96	6	89	6	86	10
Alternariolmethylether	92	8	95	4	87	11
Altersetin	78	13	185	11	144	19
Andrastin A	119	14	133	3	159	14
Apicidin	81	14	111	4	90	15
Asperphenamate	81	14	96	8	77	18
Averufin	77	15	90	6	70	19
Butenolid	95	6	51	6	48	6
Citrinin	33	15	185	9	61	19
Deoxynivalenol	79	14	69	10	54	7
Equisetin	84	15	158	5	132	17
Fumiquinazolin A	71	10	123	5	87	11
Fusarenon-X	85	6	75	6	64	7
Lotaustralin	61	6	79	4	48	6
Malformin C	90	8	101	3	91	9
Meleagrin	97	11	81	8	79	14
Moniliformin	44	5	154	3	67	7
Nivalenol	59	7	80	3	47	6
Norsolorinic acid	75	15	102	3	77	17
Patulin	83	4	82	4	68	6
Pseurotin A	91	12	129	5	117	11
Zearalenone-sulfate	84	9	116	4	98	13
Zearalenone	85	11	96	4	82	13

7 aliquot of 1 lot analysed under repeatability conditions

analyte	RE (%)	$u_{r,RE}$ (%)	SSE (%)	$u_{r,SSE}$ (%)	RA (%)	$u_{r,RA}$ (%)
15-Acetyldeoxynivalenol	105	11	62	9	64	6
15-Hydroxyculmorin	101	8	88	6	79	5
Aflatoxin B1	99	2	73	2	37	1
Aflatoxin B2	97	2	68	1	40	3
Aflatoxin G2	101	3	64	3	42	2
Aflatoxin G1	100	3	72	3	43	2
Aflatoxin M1	100	4	80	3	64	2
Brevianamid F	99	3	45	3	44	3
Cereulide	94	7	102	3	40	4
Cyclopenin	103	3	75	2	58	2
Cytochalasin J	101	4	77	3	45	4
Destruxin A	100	4	80	3	60	1
Diacetoxyscirpenol	104	1	89	2	67	2
Enniatin B	0	0	0	0	0	0
Ergocristinine	90	4	82	2	43	5
Ergocristine	99	10	66	6	48	7
Ergotaminin	85	4	61	1	39	4
Ergotamine	127	7	65	3	66	5
Fumagillin	88	5	71	4	45	6
Fumigaclavine C	102	3	75	2	63	2
Fumonisin B1	66	3	111	2	123	3
Fumonisin B2	81	3	113	2	116	2
Fumonisin B3	83	3	113	1	111	2
Gliotoxin	93	3	75	3	56	3
Griseofulvin	97	2	84	2	58	2
Herquline A	87	3	80	2	79	1
HT-2 toxin	104	7	89	5	74	2
Kojic acid	n.e.		n.e.		n.e.	
Monoacetoxyscirpenol	97	5	90	3	74	5
Mycophenolic acid	100	7	91	4	79	5
Neosolaniol	100	3	85	2	21	1
Ochratoxin A	94	2	82	2	74	3
O-Methylsterigmatocystin	97	3	79	2	57	2
Penitrem A	84	5	133	2	112	2
Phomopsin A	79	10	82	8	73	7
Questiomycin A	102	4	76	3	60	7
Quinolactacin A	96	1	82	2	68	7
Roquefortine C	86	9	56	6	39	7
Secalonic acid D	91	7	101	3	77	2
Sterigmatocystin	89	2	83	3	61	1
T-2 toxin	99	4	89	2	72	1
T2-Triol	100	18	91	16	77	8
3-Acetyldeoxynivalenol	105	5	60	4	52	5
3-Nitropropionic acid	98	1	57	1	78	2
alpha-Zearalenol	100	3	80	2	59	2
Alternariol	91	4	67	2	38	4
Alternariolmethylether	88	3	81	1	72	2
Altersetin	94	8	160	2	163	2
Andrastin A	93	3	88	2	67	2
Apicidin	92	5	94	5	126	3
Asperphenamate	92	6	84	4	93	4
Averufin	78	4	99	2	101	2
Butenolid	103	1	46	1	102	1
Citrinin	36	4	113	2	120	1
Deoxynivalenol	92	3	75	3	112	2
Equisetin	90	6	141	3	167	1
Fumiquinazolin A	86	2	86	4	109	3
Fusarenon-X	98	2	74	3	78	3
Lotaustralin	66	2	83	3	88	1
Malformin C	100	3	78	3	93	3
Meleagrin	95	5	36	3	66	4
Moniliformin	42	1	126	2	140	1
Nivalenol	62	4	92	4	86	2
Norsolorinic acid	82	10	95	2	124	1
Patulin	95	4	80	5	91	3
Pseurotin A	102	6	108	4	165	3
Zearalenone-sulfate	93	3	87	3	92	2
Zearalenone	93	2	87	1	70	2

1 aliquot of 7 different lots (within laboratory precision $u_{r,wL}$)

analyte	RA_{wL} (%)	$u_{r,wL}$ (%)
15-Acetyldeoxynivalenol	61	11
15-Hydroxyculmorin	99	17
Aflatoxin B1	75	11
Aflatoxin B2	71	10
Aflatoxin G2	69	10
Aflatoxin G1	75	10
Aflatoxin M1	81	12
Brevianamid F	54	12
Cereulide	79	24
Cyclopenin	84	13
Cytochalasin J	83	10
Destruxin A	84	7
Diacetoxyscirpenol	90	10
Enniatin B	76	21
Ergocristinine	69	10
Ergocristine	64	8
Ergotaminin	n.e.	
Ergotamine	50	9
Fumagillin	63	9
Fumigaclavine C	81	7
Fumonisin B1	69	15
Fumonisin B2	81	16
Fumonisin B3	89	17
Gliotoxin	74	12
Griseofulvin	83	9
Herquline A	73	14
HT-2 toxin	91	8
Kojic acid	n.e.	
Monoacetoxyscirpenol	92	13
Mycophenolic acid	92	13
Neosolaniol	89	8
Ochratoxin A	86	13
O-Methylsterigmatocystin	84	12
Penitrem A	95	22
Phomopsin A	70	18
Questiomycin A	96	15
Quinolactacin A	82	11
Roquefortine C	55	7
Secalonic acid D	82	14
Sterigmatocystin	72	11
T-2 toxin	83	9
T2-Triol	98	11
3-Acetyldeoxynivalenol	60	10
3-Nitropropionic acid	60	13
alpha-Zearalenol	74	7
Alternariol	78	6
Alternariolmethylether	78	6
Altersetin	121	21
Andrastin A	78	15
Apicidin	78	21
Asperphenamate	68	14
Averufin	62	11
Butenolid	41	18
Citrinin	45	13
Deoxynivalenol	66	8
Equisetin	114	20
Fumiquinazolin A	80	13
Fusarenon-X	75	8
Lotaustralin	54	10
Malformin C	75	6
Meleagrin	76	11
Moniliformin	59	15
Nivalenol	57	8
Norsolorinic acid	66	13
Patulin	69	13
Pseurotin A	111	14
Zearalenone-sulfate	87	12
Zearalenone	75	8

n.e.: not evaluated

Table S5

Table 5: Evaluation of the performance parameter RE, SSE ,RA nd their associated relative standard uncertainties (ur,RE, ur,SSE, ur,RA) for 66 mycotoxins maize.

1 aliquot of 7 different lots analysed under repeatability conditions

analyte	RE (%)	$u_{r,RE}$ (%)	SSE (%)	$u_{r,SSE}$ (%)	RA (%)	$u_{r,RA}$ (%)
15-Acetyldeoxynivalenol	95	6	101	6	95	4
15-Hydroxyculmorin	102	16	131	20	132	24
Aflatoxin B1	94	7	60	16	57	21
Aflatoxin B2	96	5	65	17	62	15
Aflatoxin G2	97	5	70	14	68	14
Aflatoxin G1	97	5	72	12	70	10
Aflatoxin M1	97	4	88	6	86	7
Brevianamid F	95	3	85	9	81	9
Cereulide	65	48	92	12	62	53
Cyclopenin	96	3	93	8	90	6
Cytochalasin J	93	6	86	11	80	9
Destruxin A	98	4	84	13	82	12
Diacetoxyscirpenol	95	4	80	12	77	14
Enniatin B	n.e.		n.e.		n.e.	
Ergocristinine	90	15	66	26	60	35
Ergocristine	89	6	55	15	49	20
Ergotaminin	86	13	57	20	49	18
Ergotamine	96	13	78	10	74	20
Fumagillin	83	8	66	22	55	21
Fumigaclavine C	101	5	75	11	75	8
Fumonisin B1	53	9	105	3	55	9
Fumonisin B2	59	6	103	2	61	7
Fumonisin B3	63	7	104	4	66	9
Gliotoxin	70	17	98	9	69	22
Griseofulvin	98	3	84	14	82	12
Herquline A	92	6	106	6	97	7
HT-2 toxin	91	15	101	18	91	10
Kojic acid	86	3	116	4	99	4
Monoacetoxyscirpenol	95	7	92	10	87	10
Mycophenolic acid	98	3	108	6	105	6
Neosolaniol	95	4	57	30	54	32
Ochratoxin A	90	3	96	3	86	3
O-Methylsterigmatocystin	86	11	87	11	75	20
Penitrem A	81	5	188	12	152	14
Phomopsin A	79	16	86	9	67	15
Questiomycin A	93	6	95	13	88	10
Quinolactacin A	94	2	92	8	87	7
Roquefortine C	n.e.		n.e.		n.e.	
Secalonic acid D	99	3	96	10	95	12
Sterigmatocystin	103	6	104	4	107	7
T-2 toxin	102	5	88	6	89	5
T2-Triol	93	26	101	16	91	20
3-Acetyldeoxynivalenol	98	6	70	11	68	13
3-Nitropropionic acid	77	11	95	12	74	19
alpha-Zearalenol	87	13	81	9	71	20
Alternariol	58	38	81	19	48	47
Alternariolmethylether	96	7	107	7	103	11
Altersetin	95	7	200	8	190	5
Andrastin A	74	31	69	26	50	39
Apicidin	91	4	142	10	128	8
Asperphenamate	98	14	132	9	129	17
Averufin	88	5	96	8	84	10
Butenolid	79	10	118	41	91	28
Citrinin	19	51	157	12	30	51
Deoxynivalenol	93	5	125	8	116	9
Equisetin	104	9	222	19	230	19
Fumiquinazolin A	88	9	169	8	147	10
Fusarenon-X	91	5	94	8	85	7
Lotaustralin	90	2	102	5	92	6
Malformin C	94	4	118	4	111	4
Meleagrin	100	8	150	5	151	8
Moniliformin	64	14	139	15	89	26
Nivalenol	88	9	93	22	84	33
Norsolorinic acid	79	10	83	5	66	11
Patulin	n.e.		n.e.		n.e.	
Pseurotin A	93	10	244	10	225	11
Zearalenone-sulfate	73	6	139	6	102	10
Zearalenone	90	9	90	12	81	10

7 aliquot of 1 lot analysed under repeatability conditions

analyte	RE (%)	$u_{r,RE}$ (%)	SSE (%)	$u_{r,SSE}$ (%)	RA (%)	$u_{r,RA}$ (%)
15-Acetyldeoxynivalenol	105	9	63	8	67	6
15-Hydroxyculmorin	104	7	82	6	82	5
Aflatoxin B1	80	4	37	3	29	3
Aflatoxin B2	94	4	39	3	37	2
Aflatoxin G2	99	5	42	2	41	4
Aflatoxin G1	99	5	43	3	43	4
Aflatoxin M1	97	2	65	2	63	3
Brevianamid F	94	5	45	4	41	4
Cereulide	41	14	41	5	17	13
Cyclopenin	99	3	60	4	58	3
Cytochalasin J	98	6	47	2	45	4
Destruxin A	101	2	60	2	61	3
Diacetoxyscirpenol	96	3	67	2	64	2
Enniatin B	n.e.		n.e.		n.e.	
Ergocristinine	68	11	43	4	29	9
Ergocristine	74	12	47	9	36	7
Ergotaminin	60	7	40	7	23	4
Ergotamine	122	8	65	6	81	6
Fumagillin	71	11	45	6	31	7
Fumigaclavine C	101	5	63	3	64	4
Fumonisin B1	66	3	125	4	81	4
Fumonisin B2	69	2	116	3	80	2
Fumonisin B3	76	2	112	3	84	1
Gliotoxin	30	6	57	4	17	4
Griseofulvin	99	3	59	2	57	2
Herquline A	90	3	79	2	72	3
HT-2 toxin	94	8	73	5	69	6
Kojic acid	72	4	82	3	61	5
Monoacetoxyscirpenol	85	7	78	7	62	6
Mycophenolic acid	103	5	76	4	81	3
Neosolaniol	102	3	21	2	21	2
Ochratoxin A	95	4	73	3	70	3
O-Methylsterigmatocystin	81	3	56	3	46	2
Penitrem A	80	4	109	3	90	3
Phomopsin A	84	9	75	11	61	6
Questiomycin A	96	8	58	10	57	5
Quinolactacin A	95	4	69	3	64	3
Roquefortine C	n.e.		n.e.		n.e.	
Secalonic acid D	80	4	76	4	62	3
Sterigmatocystin	58	4	61	2	35	4
T-2 toxin	99	3	75	3	72	3
T2-Triol	85	17	74	12	65	14
3-Acetyldeoxynivalenol	96	6	52	6	50	2
3-Nitropropionic acid	73	3	79	1	57	3
alpha-Zearalenol	66	6	61	3	39	4
Alternariol	53	3	34	5	20	3
Alternariolmethylether	80	4	73	2	57	4
Altersetin	92	3	169	3	150	2
Andrastin A	65	4	66	3	44	3
Apicidin	89	4	123	5	111	5
Asperphenamate	99	8	98	5	92	5
Averufin	81	4	106	2	82	3
Butenolid	84	4	104	1	86	5
Citrinin	25	5	124	1	29	6
Deoxynivalenol	102	6	112	2	115	6
Equisetin	65	3	169	1	108	3
Fumiquinazolin A	75	5	109	5	82	4
Fusarenon-X	91	5	79	4	71	4
Lotaustralin	91	2	86	1	79	2
Malformin C	78	4	93	2	73	2
Meleagrin	93	4	64	6	61	5
Moniliformin	64	6	142	1	90	6
Nivalenol	87	2	86	3	75	2
Norsolorinic acid	53	5	129	2	66	5
Patulin	n.e.		n.e.		n.e.	0
Pseurotin A	86	5	166	4	143	4
Zearalenone-sulfate	83	4	91	3	76	3
Zearalenone	85	2	70	2	60	2

1 aliquot of 7 different lots (within laboratory precision $u_{r,wL}$)

analyte	RA_{wL} (%)	$u_{r,wL}$ (%)
15-Acetyldeoxynivalenol	73	9
15-Hydroxyculmorin	119	23
Aflatoxin B1	35	7
Aflatoxin B2	46	7
Aflatoxin G2	50	7
Aflatoxin G1	51	8
Aflatoxin M1	n.e.	
Brevianamid F	51	8
Cereulide	62	33
Cyclopenin	67	6
Cytochalasin J	50	6
Destruxin A	75	5
Diacetoxyscirpenol	70	5
Enniatin B	73	8
Ergocristinine	37	11
Ergocristine	38	12
Ergotaminin	n.e.	
Ergotamine	31	17
Fumagillin	60	11
Fumigaclavine C	81	8
Fumonisin B1	73	20
Fumonisin B2	76	13
Fumonisin B3	81	17
Gliotoxin	25	36
Griseofulvin	63	6
Herquline A	73	10
HT-2 toxin	72	9
Kojic acid	82	17
Monoacetoxyscirpenol	72	10
Mycophenolic acid	89	9
Neosolaniol	31	10
Ochratoxin A	84	7
O-Methylsterigmatocystin	61	8
Penitrem A	107	15
Phomopsin A	75	13
Questiomycin A	79	6
Quinolactacin A	73	5
Roquefortine C	35	10
Secalonic acid D	86	9
Sterigmatocystin	48	9
T-2 toxin	82	8
T2-Triol	72	22
3-Acetyldeoxynivalenol	61	13
3-Nitropropionic acid	55	11
alpha-Zearalenol	56	7
Alternariol	35	9
Alternariolmethylether	88	7
Altersetin	183	16
Andrastin A	57	11
Apicidin	11	9
Asperphenamate	97	9
Averufin	101	14
Butenolid	60	17
Citrinin	18	60
Deoxynivalenol	96	12
Equisetin	147	11
Fumiquinazolin A	103	9
Fusarenon-X	76	8
Lotaustralin	87	16
Malformin C	76	14
Meleagrin	102	15
Moniliformin	77	18
Nivalenol	65	10
Norsolorinic acid	118	9
Patulin	n.e.	
Pseurotin A	167	21
Zearalenone-sulfate	81	10
Zearalenone	74	8

n.e.: not evaluated
